# *QuickStats*: Percentage* of U.S. Adults Aged ≥18 Years Who Have Had a Flu Vaccination in the Past 12 Months,^†^ by Sex and Age Group — National Health Interview Survey,^§^ 2017

**DOI:** 10.15585/mmwr.mm6747a7

**Published:** 2018-11-30

**Figure:**
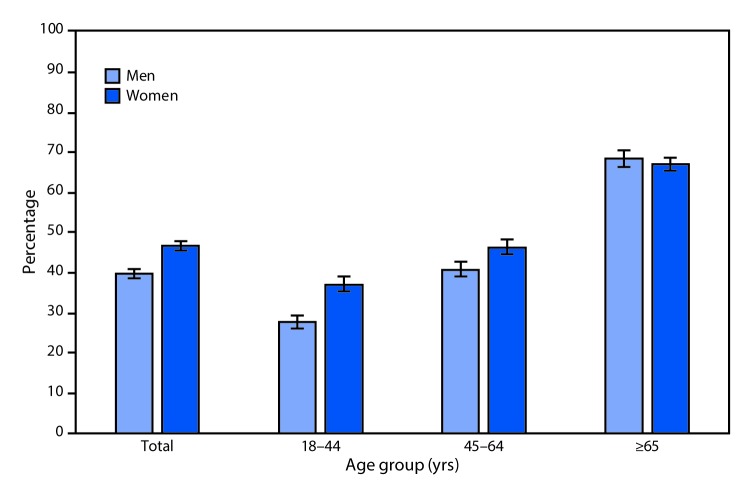
Overall, 46.7% of women and 39.9% of men aged ≥18 years have had a flu vaccination in the past 12 months. For both sexes, as age increased, a higher percentage of adults had a flu vaccination. Among men, 27.8% of those aged 18–44 years, 40.8% of those aged 45–64 years, and 68.7% of those aged ≥65 years have had a flu vaccination. Among women, 37.2% of those aged 18–44 years, 46.4% of those aged 45–64 years, and 67.1% of those aged ≥65 years have had a flu vaccination. Women aged 18–44 years and 45–64 years were significantly more likely to have had a flu vaccination compared with men of the same age groups.

**Source:** National Health Interview Survey, 2017. https://www.cdc.gov/nchs/nhis/index.htm.

